# Multimodal neuromonitoring in traumatic brain injury patients: the search for the Holy Graal

**DOI:** 10.1186/s13054-023-04679-0

**Published:** 2023-10-16

**Authors:** Fabio Silvio Taccone, Elda Diletta Sterchele, Michael Piagnerelli

**Affiliations:** 1 Department of Intensive Care, Hôpital Universitaire de Bruxelles (HUB), Université Libre de Bruxelles (ULB), Route de Lennik, 808, 1070 Brussels, Belgium; 2 Department of Intensive Care, Centre Hospitalier Universitaire (CHU)- Charleroi, Marie Curie, Université Libre de Bruxelles (ULB), Charleroi, Belgium

Dear Editor,

We recently perused the enlightening article by Svedung Wettervik et al. with great interest, a study that has provocatively questioned the established role of brain oxygenation pressure (PbtO_2_) in the intricate management of traumatic brain injury (TBI) patients [1]. The authors stated that PbtO_2_ might not serve as an optimal outcome measure to ascertain the adequacy of cerebral hemodynamics optimization. This assertion stems from several observations: (a) low PbtO_2_ values observed in only 17% of the monitoring time; (b) low PbtO_2_ rarely concurrent with high intracranial pressure (ICP), low cerebral perfusion pressure (CPP) or altered cerebral autoregulation indices (PRx); and (c) the lack of correlation between PbtO_2_ and ICP, CPP and PRx. While the article is undeniably of paramount significance and the authors have commendably delineated the study's limitations, we believe there are certain nuances and considerations that warrant further discussion for the readership, especially when broaching the topic of neuromonitoring in this clinical setting.

Firstly, this study provides empirical evidence reinforcing the notion that cerebral perfusion and cerebral oxygenation are not inextricably linked. It is imperative to understand that cerebral oxygenation is a multifaceted entity, contingent not solely on regional cerebral blood flow but also profoundly influenced by arterial oxygen content, thus encompassing both oxygen and hemoglobin levels, oxygen consumption rates and the efficiency of oxygen diffusion, which is intrinsically tied to microvascular function, which is frequently altered after TBI [2]. Given this intricate interplay, it is not surprising that a significant correlation between PbtO_2_ and the aforementioned perfusion variables remains elusive. A dissociation between global perfusion and tissue oxygenation has been already reported in septic patients [3]; as such, PbtO_2_, ICP, CPP and autoregulation assessment proffer complementary insights rather than merely echoing redundant data and should therefore be integrated into a multimodal approach to better understand the consequences of TBI on tissue perfusion and oxygenation. Consequently, clinical scenarios may arise where patients exhibit satisfactory oxygenation, as might be observed in low CPP and ICP values coupled with low oxygen consumption, or in cases marked by elevated ICP, concurrent with cerebral hyperemia.

Secondly, the precise positioning of the PbtO_2_ probe, a detail of paramount importance, was not consistently delineated in the study. Prior investigations have shown that probes strategically placed in non-injured cerebral territories might not yield clinically relevant data; specifically, the absence of a correlation between reduced PbtO_2_ values and adverse outcomes has been documented in this setting [4]. Thus, it would necessitate exceedingly elevated ICP and reduced CPP levels to compromise oxygenation in regions of the brain that ostensibly appear unaffected after TBI [4]. This finding could elucidate the consistently within-norm PbtO_2_ measurements across wide ranges of ICP and CPP values, as reported in this study [1].

Thirdly, the study was conducted under the aegis of an institutional protocol tailored to optimize PbtO_2_, but not autoregulation, in clinical practice. This approach invariably reduced the incidence of PbtO_2_ values below thresholds that were considered dangerous; however, no specific interventions were delineated for instances of compromised autoregulation. Given that PbtO_2_ can register falsely reassuring readings in the presence of high PaO_2_ values [2], integrating the PbtO_2_/PaO_2_ ratio [5] into the analytical framework might have furnished invaluable insights on the possible association between tissue hypoxia and impaired cerebral autoregulation. If the persistent dissociation between tissue oxygenation and autoregulation indices would still be established, the ultimate objective of enhancing hemodynamics in TBI patients remains uncertain. This raises the question of whether achieving optimal hemodynamics would entail optimizing autoregulation for its maximal efficacy (taking into account vascular responses to pressure or other stimuli) or improving tissue oxygen delivery, essential for cellular function.

Lastly, the study spans a notably protracted timeframe, from 2002 to 2022. It is pivotal to recognize that PbtO_2_-driven therapeutic strategies have been ushered into the clinical arena predominantly over the past decade. This evolution in clinical practice paradigms could potentially have swayed the observed associations between PbtO_2_, other pertinent variables, and overarching clinical management strategies. Incorporating time as an additional confounding factor in the subgroup analysis of the study could have helped in the interpretation of its findings.

## Data Availability

Not applicable.

## References

[1] SvedungWettervik T, Beqiri E, Bögli SY, Placek M, Guilfoyle MR, Helmy A, Lavinio A, O'Leary R, Hutchinson PJ, Smielewski P (2023). Brain tissue oxygen monitoring in traumatic brain injury: Part I-To what extent does PbtO_2_ reflect global cerebral physiology?. Crit Care.

[2] Robba C, Taccone FS, Citerio G (2022). Monitoring cerebral oxygenation in acute brain-injured patients. Intensive Care Med.

[3] Bakker J, Ince C (2020). Monitoring coherence between the macro and microcirculation in septic shock. Curr Opin Crit Care.

[4] Ponce LL, Pillai S, Cruz J, Li X, Julia H, Gopinath S, Robertson CS (2012). Position of probe determines prognostic information of brain tissue PO_2_ in severe traumatic brain injury. Neurosurgery.

[5] Dellazizzo L, Demers SP, Charbonney E, Williams V, Serri K, Albert M, Giguère JF, Laroche M, Williamson D, Bernard F (2018). Minimal PaO_2_ threshold after traumatic brain injury and clinical utility of a novel brain oxygenation ratio. J Neurosurg.

